# Inhibition of DUSP6 sensitizes ovarian cancer cells to chemotherapeutic agents via regulation of ERK signaling response genes

**Published:** 2019-05-21

**Authors:** Nicole E. James, Lindsey Beffa, Matthew T. Oliver, Ashley D. Borgstadt, Jenna B. Emerson, Clinton O. Chichester, Naohiro Yano, Richard N. Freiman, Paul A. DiSilvestro, Jennifer R. Ribeiro

**Affiliations:** ^1^ Women and Infants Hospital, Department of Obstetrics and Gynecology, Program in Women’s Oncology, Providence, RI, USA; ^2^ Department of Surgery, Roger Williams Medical Center, Providence, RI, USA; ^3^ Department of Pharmacy, University of Rhode Island, Kingston, RI, USA; ^4^ Department of Molecular and Cell Biology and Biochemistry, Brown University, Providence, RI, USA

**Keywords:** DUSP6, HE4, ovarian cancer, chemoresistance, ERK signaling

## Abstract

Dual specificity phosphatase 6 (DUSP6) is a protein phosphatase that deactivates extracellular-signal-regulated kinase (ERK). Since the ovarian cancer biomarker human epididymis protein 4 (HE4) interacts with the ERK pathway, we sought to determine the relationship between DUSP6 and HE4 and elucidate DUSP6’s role in epithelial ovarian cancer (EOC). Viability assays revealed a significant decrease in cell viability with pharmacological inhibition of DUSP6 using (E/Z)-BCI hydrochloride in ovarian cancer cells treated with carboplatin or paclitaxel, compared to treatment with either agent alone. Quantitative PCR was used to evaluate levels of ERK pathway response genes to BCI in combination with recombinant HE4 (rHE4), carboplatin, and paclitaxel. Expression of EGR1, a promoter of apoptosis, was higher in cells co-treated with BCI and paclitaxel or carboplatin than in cells treated with chemotherapeutic agents alone, while expression of the proto-oncogene c-JUN was decreased with co-treatment. The effect of BCI on the expression of these two genes opposed that of rHE4. Pathway focused quantitative PCR also revealed suppression of *ERBB3* in cells co-treated with BCI plus carboplatin or paclitaxel. Finally, expression levels of DUSP6 in EOC tissue were evaluated by immunohistochemistry, revealing significantly increased levels of DUSP6 in serous EOC tissue compared to adjacent normal tissue. A positive correlation between HE4 and DUSP6 levels was determined by Spearman Rank correlation. In conclusion, DUSP6 inhibition sensitizes ovarian cancer cells to chemotherapeutic agents and alters gene expression of ERK response genes, suggesting that DUSP6 could plausibly function as a novel therapeutic target to reduce chemoresistance in EOC.

## INTRODUCTION

Ovarian cancer remains the most common and deadly gynecologic cancer, responsible for 240,000 diagnoses and 152,000 deaths worldwide each year [1]. The 5-year survival rate remains at 35% [2], which is largely due to difficulty with early diagnosis, coupled with the frequency of chemoresistant recurrences [3]. A majority of epithelial ovarian cancer (EOC), the most common subtype of ovarian cancer, is initially responsive to chemotherapy. However, once the disease recurs, chemoresistance inevitably develops and the patient eventually will succumb to their illness [4]. Therefore, there is a need for improved diagnostic approaches, as well as novel treatment targets to combat chemoresistance.

Human epididymis protein 4 (HE4) has been established as a novel clinical biomarker for EOC. Inclusion of preoperative serum HE4 levels into the diagnostic Risk of Ovarian Malignancy Algorithm (ROMA) results in demonstrably improved specificity and sensitivity in detection and monitoring of the disease over Cancer Antigen 125 (CA 125), pelvic sonography, and menopausal status alone [5]. Research has also shown its ability to support EOC pathogenesis, including the promotion of proliferation, chemoresistance, anti-estrogen resistance, adhesion, invasion, and migration [6–17]. One oncogenic pathway that has consistently been shown to interact with HE4 in several studies is the extracellular signal regulated kinase (ERK) pathway. Several reports indicate that ERK activation is enhanced with HE4 treatment or overexpression, while ERK activation is conversely reduced with HE4 knockdown [9, 15, 16]. Our lab has revealed a complex response of ERK to recombinant HE4 treatment; specifically, we have observed downregulation of ERK phosphorylation at early time points following treatment with recombinant HE4, and its upregulation at later time points [9]. However, the precise nature of HE4’s interaction with the ERK pathway is not known in the context of EOC.

Dual specificity phosphatase 6 (DUSP6) is a key negative regulator of ERK signaling via dephosphorylation of ERK at serine/tyrosine residues. ERK activation upregulates gene expression of DUSP6, which promotes a negative feedback loop that suppresses ERK activation [18]. DUSP6 has differing effects on tumor progression depending on the tumor type. In pancreatic and lung cancer, DUSP6 is considered a tumor suppressor [19, 20]. However, in glioblastoma and HER-2 positive breast cancer, it is upregulated and considered oncogenic [21, 22]. In gastric cancer, DUSP6 inhibition can promote chemosensitivity [23], and it has also been characterized as a therapeutic target in acute lymphoblastic leukemia [24]. Although one ovarian cancer study suggested that DUSP6 acts as a tumor suppressor [25], the goal of the present study was to determine the potential relationship between HE4 and DUSP6 in the context of EOC.

## RESULTS

### HE4 regulates DUSP6 levels in ovarian cancer cells

**Figure 1:**
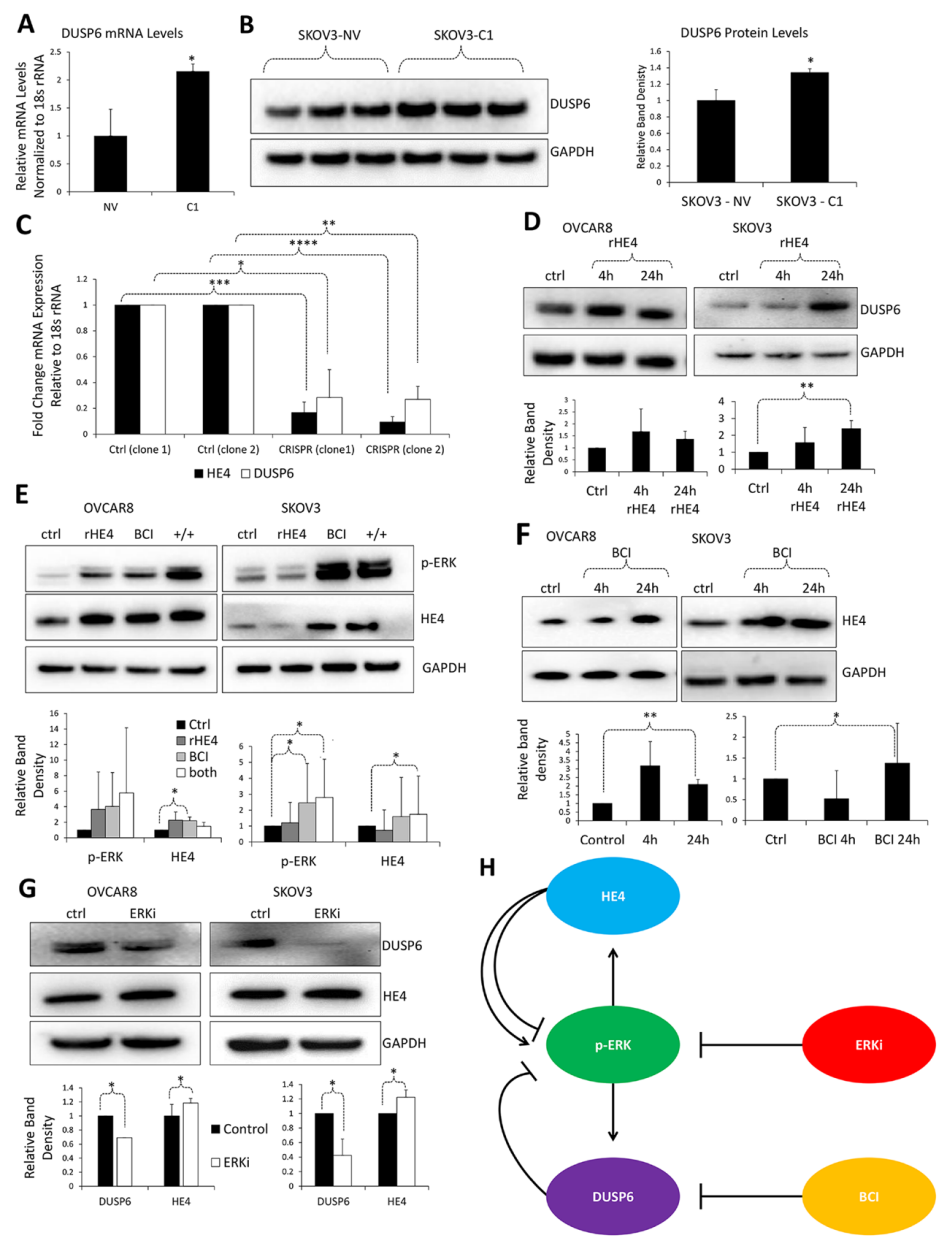
HE4 regulates DUSP6 levels in ovarian cancer cells **(A)**. qPCR revealed upregulation of DUSP6 mRNA levels in SKOV3 cells overexpressing HE4 (C1) compared to null vector (NV) counterparts. **(B)** Western blot indicated that DUSP6 levels were upregulated in SKOV3-C1 cells compared to SKOV3-NV cells. **(C)** qPCR revealed downregulation of DUSP6 mRNA levels in two unique clones of SKOV3 cells with stable CRISPR-mediated HE4 knockdown. **(D)** Western blot showed that DUSP6 levels were upregulated with rHE4 treatment in OVCAR8 and SKOV3 cells. **(E)** Western blot showed that BCI treatment increased HE4 and activated ERK levels (+/+ refers to cells co-treated with BCI and rHE4). **(F)** Western blot showed that BCI increased phospho-ERK and HE4 levels in OVCAR8 and SKOV3 cells. **(G)** Western blot revealed that ERK inhibition downregulated DUSP6 and slightly increased HE4 levels. **(H)** Working model for interaction of HE4 and DUSP6 with the ERK pathway. Activated ERK is known to upregulate DUSP6 mRNA; DUSP6 then suppresses ERK activation in a negative feedback loop, while the DUSP6 inhibitor BCI reverses this effect. Also as expected, inhibition of the ERK pathway downregulated DUSP6 levels; contrary to expectations, a slight increase was seen in HE4 levels. However, phospho-ERK levels appear to positively associate with HE4, suggesting a positive relationship between these proteins. This idea is reinforced by studies showing that HE4 upregulates phospho-ERK, which may occur in a time- or context-dependent manner. Error bars for all graphs represent standard deviation of ≥3 biological replicates. ^*^p<0.05, ^**^p<0.005, ^***^p<0.0005, ^****^p<0.00005.

To begin to elucidate the relationship between HE4 and DUSP6, we examined DUSP6 mRNA and protein levels in SKOV3 ovarian cancer cells overexpressing HE4 (SKOV3-C1) compared to their null vector (NV) controls, which express low levels of HE4 naturally. These assays revealed increased DUSP6 mRNA (2.15-fold, p=0.008) and protein (1.34-fold, p=0.007) in HE4 overexpressing SKOV3 cells (Figure 1A-1B). To determine if this upregulation of DUSP6 was dependent upon continued expression of HE4, HE4 was knocked down in SKOV3-C1 cells using CRISPR/Cas9. As expected, DUSP6 levels were concomitantly reduced in two independent knockdown clones (0.28-fold and 0.27–fold, p=0.015 and p=9.2×10^-4^, respectively) (Figure 1C). Finally, treatment with recombinant HE4 (rHE4) at 4h and 24h increased DUSP6 levels in OVCAR8 (1.68-fold, p=ns, 1.37-fold, p=ns) and SKOV3 cells (1.57-fold, p=ns, 2.39-fold, p=0.032)(Figure 1D). These results indicate that HE4 promotes DUSP6 expression in two human ovarian cancer cell lines.

Because both DUSP6 and HE4 are involved in ERK signaling, we next examined ERK phosphorylation in response to rHE4 treatment or DUSP6 inhibition with BCI, a cell permeable allosteric inhibitor of DUSP6 MAPK phosphatase activity. Interestingly, the upregulation of phospho-ERK by rHE4 was only observed sporadically in OVCAR8 and SKOV3 cells at 24 h (Figure 1E). We and others have reported activation of ERK by HE4 [9, 15, 16]; specifically, we found that rHE4 suppressed phospho-ERK levels at 1 and 4 h but increased them at 24 and 48 h [9]. While we did not perform a time course in the present study, the inconsistent results in this study suggest that the exact timing of phospho-ERK regulation by rHE4 may be variable.

Next, we observed an increase in phospho-ERK with BCI treatment in OVCAR8 and SKOV3 (4.03-fold and 2.45-fold, p=n/s and p=0.043, respectively), as would be expected if the negative regulation of ERK by DUSP6 is being suppressed (Figure 1E). Finally, co-treatment with BCI and rHE4 resulted in upregulation of phospho-ERK in OVCAR8 and SKOV3 (5.78-fold and 2.80-fold, p= n/s and p=0.019, respectively). In addition, there was a general positive relationship between phospho-ERK and HE4 levels, corroborating a positive relationship between activation of ERK signaling and intracellular HE4 levels (Figure 1E). We further confirmed an upregulation of HE4 by BCI at 24 h in OVCAR8 and SKOV3 (2.05-fold, p=.001, 2.28-fold, p=0.041) (Figure 1F).

Furthermore, we determined the effect of 2-(2-Chloro-4-iodophenylamino)-N-cyclopropylmethoxy-3,4-difluorobenzamide, a highly specific inhibitor of MKK1 and the ERK pathway, on DUSP6 and HE4 levels. ERK inhibition downregulated DUSP6 in OVCAR8 and SKOV3 cells, which was expected since ERK is known to positively regulate DUSP6 expression to create a negative feedback loop on ERK activation (0.69-fold and 0.42–fold, p=0.016 and p=0.006, respectively). In addition, a small but significant increase in HE4 levels was also observed in OVCAR8 and SKOV3 cells treated with the ERK inhibitor (1.18-fold and 1.22–fold, p=0.004 and p=0.009, respectively) (Figure 1G). The upregulation of HE4 with ERK inhibition was contrary to our expectations that ERK inhibition would suppress HE4 levels, since we observed in a previous study that cells treated with recombinant human EGF increased HE4 levels following ERK activation [14]. We hypothesize that there may be a compensatory upregulation of HE4 via other mechanisms following ERK inhibition. Collectively, these results highlight that HE4 positively regulates DUSP6 levels in ovarian cancer cells, and both proteins regulate ERK signaling in time-dependent manners. A working model for the relationship between HE4, DUSP6, and ERK signaling is outlined in Figure 1H. Triplicate western blots from which statistics were obtained can be seen in Supplementary Figure 1.

### Inhibition of DUSP6 sensitizes ovarian cancer cells to chemotherapeutic drugs

Next, we sought to determine the function of DUSP6 in ovarian cancer cells. Since HE4 is known to promote chemosresistance *in vitro*, and HE4 is associated with poor chemoresponse in EOC patients [9], we treated SKOV3 and OVCAR8 cells with a DUSP6 inhibitor (BCI) alone or in combination with paclitaxel or carboplatin, the standard of care chemotherapeutic agents in EOC. A dose response curve was generated to determine an optimal dose of BCI for all subsequent experiments (Supplementary Figure 2).

Treatment of cells with 3.75 µM BCI alone resulted in a small but significant reduction in cell viability relative to control as determined by 3-(4,5-dimethylthiazol-2-yl)-5-(3-carboxymethoxyphenyl)-2-(4-sulfophenyl)-2H-tetrazolium) (MTS) assay – 86.3% (p=0.007) and 84.7% (p=.004) in OVCAR8 and SKOV3, respectively. In both cell lines, co-treatment with BCI and 100 µM carboplatin resulted in a statistically significant synergistic effect on cytotoxicity compared to either treatment alone. Carboplatin alone treatment resulted in 88.8% (p=0.002) and 86.8% (p=4.9×10^-4^) survival in OVCAR8 and SKOV3 cells, respectively, while BCI with carboplatin resulted in 33.3% (p=2.51×10^-7^) and 50.1% (p=5.46×10^-7^) survival in OVCAR8 and SKOV3 cells, respectively. The difference between carboplatin alone and carboplatin with BCI was also statistically significant in OVCAR8 and SKOV3 (p=1.78×10^-7^ and 3.25×10^-6^, respectively).

**Figure 2:**
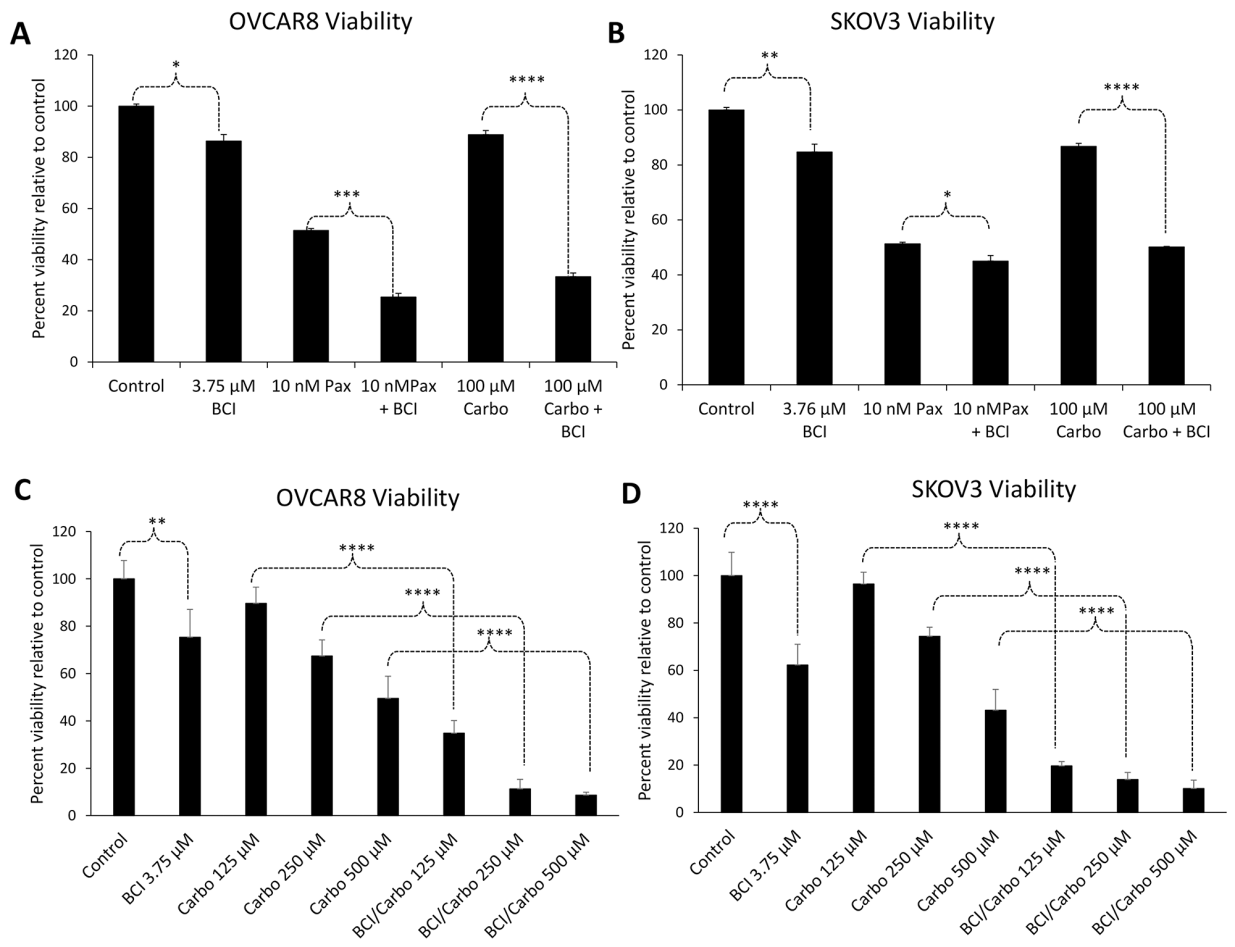
Inhibition of DUSP6 sensitizes ovarian cancer cells to chemotherapeutic drugs. OVCAR8 cells **(A)** and SKOV3 cells **(B)** exhibited reduced viability by MTS assay when co-treated for 24 h with the DUSP6 inhibitor BCI and either paclitaxel or carboplatin compared to either chemotherapeutic agent alone. OVCAR8 cells **(C)** and SKOV3 cells **(D)** were treated with various doses of carboplatin alone or in combination with 3.75 uM BCI for 24 h. Cell viability was measured with MTS assay. A synergistic effect was observed with the addition of BCI to carboplatin at all doses tested. Error bars represent standard deviation from ≥3 biological replicates. For clarity, only significance between Ctrl vs. BCI and single agent chemo vs. BCI+chemo are indicated on the graph. Error bars represent standard deviation of ≥3 biological replicates. ^*^p<0.05, ^**^p<0.005; ^***^p<.0005, ^****^p<.00005.

Likewise, significantly more death was noted with BCI and 10 nM paclitaxel treatment, with survival of 51.4% in OVCAR8 (p=1.73×10^-6^) and 51.3% in SKOV3 (p=9.37×10^-7^) with paclitaxel alone, and 25.4% (p=1.8×10^-6^) and 45.0% (p=9.66×10^-7^) with the addition of BCI with paclitaxel. The difference between paclitaxel alone and paclitaxel with BCI was statistically significant in OVCAR8 and SKOV3 (p=1.1×10^-4^ and 0.022, respectively) (Figure 2A-2B). In order to verify the results at multiple doses of carboplatin, cells were treated with 3.75 uM BCI with varying doses of carboplatin (100, 250, 500 µM). At all doses tested, combinatorial treatment with BCI and carboplatin resulted in significantly reduced viability of OVCAR8 and SKOV3 cells compared to carboplatin alone (Figure 2C-2D).

### DUSP6 inhibition alters expression of ERK pathway responsive genes

In order to determine how regulation of ERK signaling by BCI versus rHE4 might affect downstream gene expression, we treated cells with BCI alone or in combination with rHE4, paclitaxel, or carboplatin, and examined expression of the ERK pathway response genes *EGR1* and c-*JUN*. EGR1 is a transcription factor involved in promoting apoptosis in many cancers [26–29], and is involved in cisplatin resistance in esophageal and ovarian cancer cells [29–30]. We have previously shown that HE4 suppresses cisplatin-induced *EGR1* gene upregulation in SKOV3 cells [9]. On the other hand, c-*JUN* is a transcription factor involved in promoting cell survival and growth, and is associated with resistance to platinum-based chemotherapy [31].

**Figure 3:**
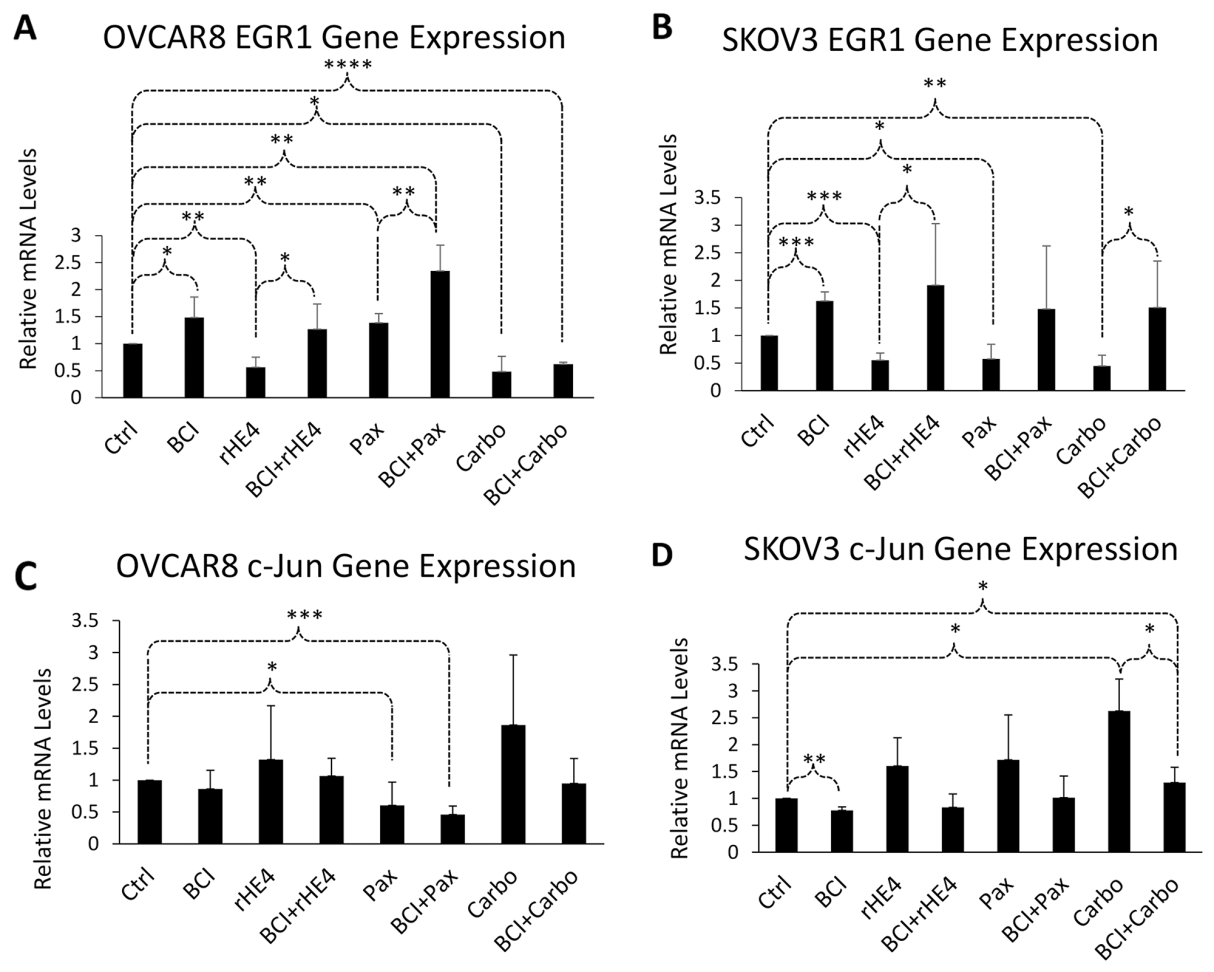
DUSP6 inhibition alters expression of ERK pathway responsive genes. OVCAR8 and SKOV3 cells were treated with BCI, carboplatin, BCI+carboplatin, paclitaxel, or BCI+paclitaxel for 24 h and qPCR was performed. **(A)** BCI opposed the effect of rHE4 on *EGR1* levels in OVCAR8 cells, and *EGR1* mRNA levels were higher in cells co-treated with BCI and chemotherapeutic drugs than in cells treated with chemotherapy alone. **(B)** BCI opposed the effect of rHE4 on c-*JUN* levels in OVCAR8 cells, and c-*JUN* mRNA levels were lower in cells co-treated with BCI and chemotherapeutic drugs than in cells treated with chemotherapy alone. **(C)** BCI opposed the effect of rHE4 on *EGR1* levels in SKOV3 cells, and *EGR1* mRNA levels were higher in cells co-treated with BCI and chemotherapeutic drugs than in cells treated with chemotherapy alone. **(D)** BCI opposed the effect of rHE4 on c-*JUN* levels in SKOV3 cells, and c-*JUN* mRNA levels were lower in cells co-treated with BCI and chemotherapeutic drugs than in cells treated with chemotherapy alone. Error bars represent standard deviation of n≥3 independent experiments. ^*^p<.05; ^**^p<.005; ^***^p<.0005; ^***^p<.00005.

Treatment with BCI modestly upregulated *EGR1* expression by 1.48-fold (p=0.022) and 1.63-fold (p=1.2×10^-4^) in OVCAR8 and SKOV3 cells, respectively. Conversely, treatment with rHE4 resulted in 0.56-fold (p=0.0016) and 0.55-fold (p=2.5×10^-4^) reduced *EGR1* expression relative to control in OVCAR8 and SKOV3, respectively—a result that is in agreement with our previous study showing HE4 suppresses cisplatin-mediated upregulation of *EGR1* [9]. The effect of BCI on *EGR1* expression was more apparent with rHE4 co-treatment, where it significantly reversed the downregulation of *EGR1* by rHE4 in OVCAR8 (p=0.016) and SKOV3 (p=0.026). Furthermore, co-treatment with BCI and either paclitaxel or carboplatin upregulated expression of *EGR1* compared to treatment with either chemo drug alone. *EGR1* levels were increased by 2.35-fold with paclitaxel and BCI versus 1.38-fold with paclitaxel alone (p=0.005). Although the response was not as robust with paclitaxel, a similar trend was observed with carboplatin co-treatment (Figure 3A-3B).

Conversely, treatment with BCI resulted in 0.86-fold (n/s) and 0.78-fold (p=0.004) reduced c-*JUN* levels relative to control in OVCAR8 and SKOV3 cells, respectively, while rHE4 upregulated c-*JUN* by 1.32-fold (n/s) and 1.60-fold (n/s). Again, co-treatment with BCI and rHE4 reversed the upregulation of c-*JUN* by rHE4. We also observed a decrease in c-*JUN* levels in BCI and chemotherapy treated groups compared to chemotherapy alone groups, although only the carboplatin versus BCI/carboplatin result in SKOV3 cells reached the cutoff for significance (p=0.039; Figure 3C-3D). Collectively, these results show that BCI opposes the effects of HE4 on *EGR1* and c-*JUN* expression, and promotes *EGR1* expression while suppressing c-*JUN* expression in cells exposed to chemotherapeutic drugs.

### DUSP6 inhibition alters ovarian cancer cells’ chemotherapy response genomic profile

**Figure 4:**
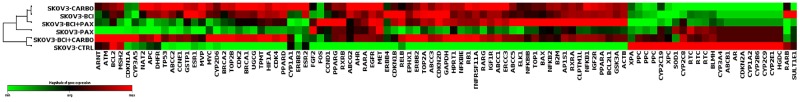
DUSP6 inhibition alters ovarian cancer cells’ chemotherapy response genomic profile. Heatmap of gene expression (analyzed by Human Cancer Drug Resistance pathway focused qPCR array) in SKOV3 cells treated with BCI, carboplatin, paclitaxel, or chemotherapy in combination with BCI. Clustering, performed by the Qiagen GeneGlobe Data Analysis Center, indicates groups with most similarity between each other.

In order to gain a further understanding of the effect of BCI combinatorial treatment on gene expression profiles, RNA from SKOV3 cells treated with vehicle, BCI, carboplatin, BCI/carboplatin, paclitaxel, and BCI/paclitaxel was used on a pathway-focused qPCR array for Human Cancer Drug Resistance (Qiagen, PAHS-004Z). The heat map represents the similarities in profiles between carboplatin and BCI. The carboplatin/BCI profile shares similarities with both the carboplatin alone or BCI alone profile. The paclitaxel gene profile stands out as unique, while BCI/paclitaxel shows a mixed expression signature between BCI alone and paclitaxel alone treatments (Figure 4). Collectively, the genomic analysis of cells treated with BCI and chemotherapy agents reveal that BCI promotes similar chemo-response and cell death pathways as carboplatin. However, a more in-depth analysis of the specific genes regulated by each of these treatments revealed differences that may contribute to the role of DUSP6 in the increased chemosensitivity of these cells.

The top five upregulated and downregulated genes from each treatment comparison are outlined in Table 1. We observed a very similar expression profile with BCI alone and carboplatin alone treatments, with the top increased mRNAs compared to control with either treatment being *ELK1, GAPDH, ERBB2, ABCC1, BCL2L1*, and *PPARD*, and the most reduced mRNAs including Cytochrome P450 family genes, as well as *CDKN2A* and *AR*. BCI/carboplatin co-treatment also produced similar effects on gene expression as carboplatin alone or BCI alone. The top genes upre- gulated in all three groups relative to control were *ELK1, GAPDH, ERBB2, ABCC1, BCL2L1*, and *PPARD*. Conversely, *ERBB3* and *RARB* were the genes most downregulated with BCI/carboplatin co-treatment compared to control, which differed from BCI alone or carboplatin alone treatments.

**Table 1:** Most differentially expressed genes from pathway focused qPCR array

Group Comparison	Gene	Fold-change (up)	Gene	Fold-change (down)
BCI vs. Ctrl	ELK1	29.2	CYP2E1	-2.42
	GAPDH	28.47	CYP1A2	-2.42
	ERBB2	23.48	CDKN2A	-2.42
	ABCC1	23.02	AR	-2.42
	BCL2L1	18.04	ABCB1	-2.42
Carbo vs. Ctrl	ABCC1	27.74	CYP3A4	-2.76
	ELK1	27.47	CYP2B6	-2.76
	GAPDH	26.35	CDKN2A	-2.76
	RARG	20.72	SULT1E1	-2.76
	ERBB2	19.01	AR	-2.76
BCI/Carbo vs. Ctrl	ELK1	33.41	ERBB3	-3.18
	GAPDH	27.79	RARB	-2.70
	ABCC1	25.27	XPA	-2.08
	ERBB2	24.92		
	PPARD	19.00		
Pax vs. Ctrl	RARA	11.16	RARB	-5.48
	CCND1	9.64	ATM	-5.19
	BCL2L1	9.46	ARNT	-3.53
	NFKB2	8.90	BCL2	-3.17
	NFKBIB	8.73	NAT2	-2.88
BCI/Pax vs. Ctrl	ELK1	44.81	RARB	-5.53
	ABCC1	25.65	XPA	-3.79
	GAPDH	25.28	APC	-2.52
	NFKBIB	24.27	CYP1A1	-2.38
	BCL2L1	21.26	MSH2	-2.13
BCI/Carbo vs. Carbo	CYP2C8	2.57	ERBB3	-15.13
	CYP3A5	2.22	RARB	-2.05
	CYP2B6	2.15		
	CYP1A2	2.15		
	CYP3A4	2.15		
BCI/Pax vs. Pax	ELK1	16.69	ERBB3	-2.07
	RELB	9.16		
	RARG	6.59		
	ERBB2	5.95		
	GAPDH	5.84		

Paclitaxel treatment alone produced a unique gene expression signature, with top genes upregulated compared to control being *RARA, CCND1, BCL2L1, NFKB2,* and *NFKBIB*. The most downregulated genes were *RARB, ATM, ARNT, BCL2*, and *NAT2*. BCI and paclitaxel co-treatment produced a mixed signature, with *ELK1, ABCC1, GAPDH, NFKBIB*, and *BCL2L1* being the most upregulated genes, and *RARB, XPA, APC, CYP1A1*, and *MSH2* being the most downregulated.

Further comparisons were performed between carboplatin or paclitaxel alone and in combination with BCI. The differences in upregulated genes between BCI/carboplatin and carboplatin alone were modest and comprised Cytochrome P450 family genes. Only two genes were downregulated in BCI/carboplatin versus carboplatin alone, and of those, only *ERBB3* showed robust downregulation. The differences in upregulated genes between paclitaxel/BCI and paclitaxel alone reflected the addition of BCI to paclitaxel. There was robust upregulation of *ELK1, ERBB2, RARG, GAPDH*, and *RELB* with co-treatment, which was not noted with paclitaxel treatment alone, as well as modest downregulation of *ERBB3*. A similar suppression of *ERBB3* was observed in OVCAR8 cells treated with BCI and chemotherapeutic drugs (Supplementary Figure 3). The complete results of the qPCR array can be found in Supplementary Dataset 1.

### DUSP6 levels are upregulated in EOC tissue compared to adjacent normal tissue, and correlate with HE4 tissue levels

**Figure 5:**
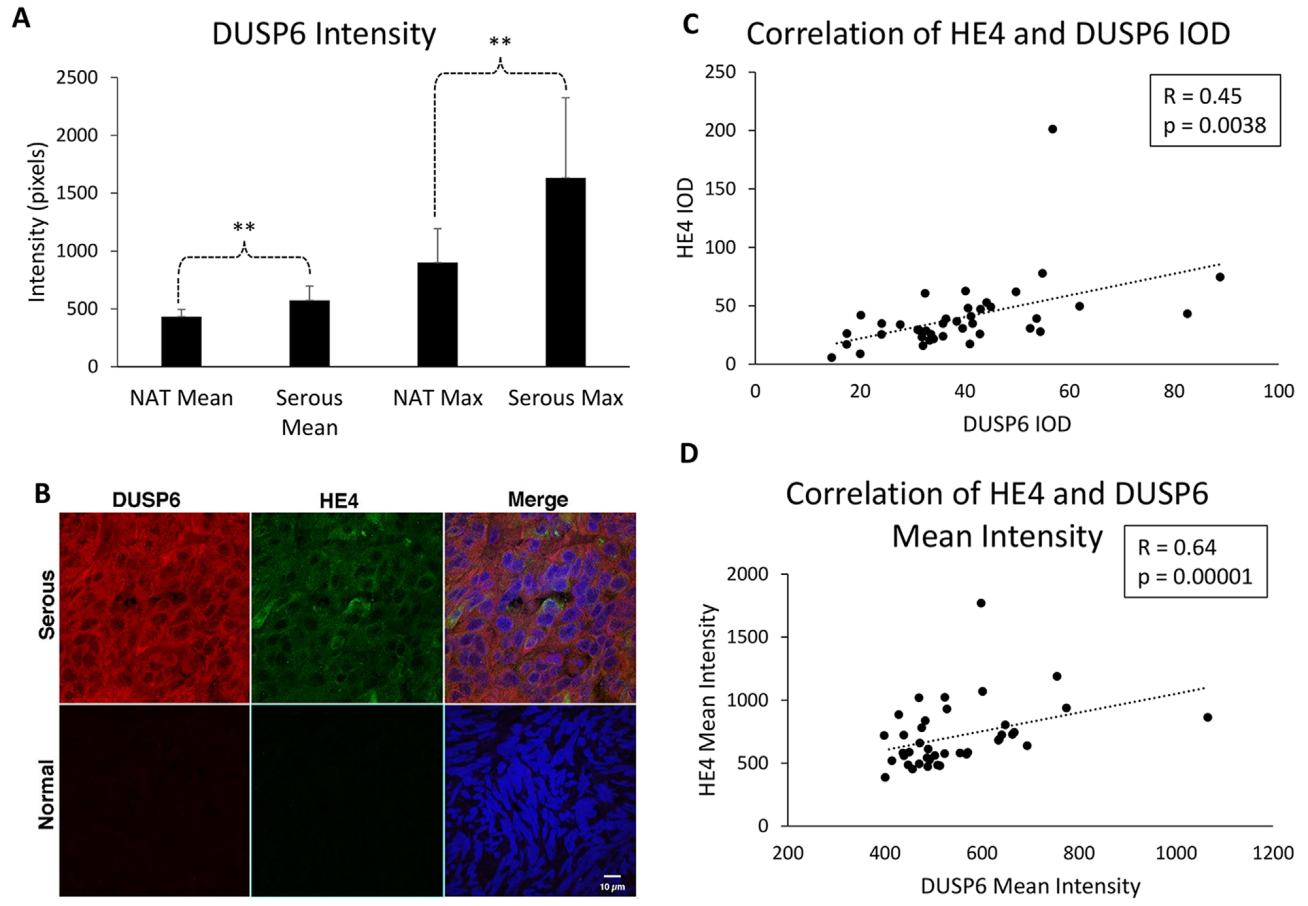
DUSP6 levels are higher in EOC tissue than normal adjacent tissue, and correlate with HE4 tissue levels **(A)**. DUSP6 mean and maximum intensity staining is higher in serous EOC tissue (n=40) than in normal adjacent tissue (NAT) (n=7). Error bars represent standard deviation. ^**^p<0.005 **(B)** Representative images of NAT and serous EOC DUSP6 staining. **(C)** Correlation of DUSP6 and HE4 mean intensity. **(D)** Correlation of DUSP6 and HE4 integrated optical density (IOD).

To verify the clinical relevance of our findings, we performed immunohistochemistry of DUSP6 in an EOC tumor microarray and compared levels in serous adenocarcinoma samples (n=40) to levels in normal adjacent tissue (NAT; n=7). Mean intensity of DUSP6 was 573 (+/− 19.6) in EOC samples, and 432 (+/−24.5) in NAT (p=0.0027). Moreover, maximal intensity was significantly greater in serous EOC samples than NAT. Maximum intensity was 1632 (+/−109.6) for EOC and 900 (+/−110.3) for NAT (p=0.0045), indicating that some areas of EOC exhibited particularly strong staining for DUSP6 (Figure 5A). Representative images are shown in Figure 5B.

In order to determine if a correlation exists between HE4 levels and DUSP6 levels in EOC, we co-stained for both proteins in the ovarian tissue microarray, and calculated correlations for mean intensity values and integrated optical density (IOD). Spearman Rank correlation test revealed a positive correlation between DUSP6 and HE4 mean intensities (R=0.45, p=0.0038) and IOD values (R=0.64, p=1.0×10^-5^) (Figure 5C-5D). Together, these results suggest that DUSP6 may be involved in promoting tumorigenesis and chemoresistance in EOC, and highlight a relationship between HE4 and DUSP6 *in vitro* and in EOC.

## DISCUSSION

In this study, we found that HE4 promotes upregulation of DUSP6 in ovarian cancer cells, and that levels of these proteins positively correlate in EOC tissue. We also determined that inhibition of DUSP6 promotes chemosensitivity of two different ovarian cancer cell lines, which coincided with increased expression of pro-apoptotic *EGR1*, and reduced expression of oncogenic c-*JUN*. Lastly, we examined the genomic signature of cells treated with a DUSP6 inhibitor alone or in combination with the chemotherapeutic drugs carboplatin or paclitaxel, and found a reduction in *ERBB3* mRNA with BCI and chemotherapy co-treatment compared to chemotherapy alone.

The nature of the relationship between HE4 and DUSP6 was revealed by examining the effect of DUSP6 inhibition and ERK inhibition on HE4 and DUSP6 levels. Counter to our expectations, inhibition of DUSP6 with BCI actually resulted in an upregulation of HE4. This may be explained by BCI-mediated upregulation of phospho-ERK leading to an upregulation of HE4, since our previous published data showed that activation of the epidermal growth factor receptor (EGFR) signaling pathway leads to an increase in HE4 [14]. However, it does highlight the fact that activation of phospho-ERK via different mechanisms may produce differing effects on both gene expression and cell behavior. Moreover, while ERK inhibition produced an expected suppression of DUSP6 levels, it actually increased HE4 levels, again highlighting an as yet unexplained relationship between DUSP6, HE4, and ERK signaling. There are likely many time- and context-dependent pathways and feedback mechanisms that play a role in the regulation of HE4 and DUSP6.

The two ERK responsive genes we characterized show opposite expression patterns with BCI treatment. *EGR1* is activated by ERK via the transcription factor ELK-1, and EGR1 is itself a transcription factor that activates expression of genes regulating proliferation, differentiation, and apoptosis [32, 33]. A previous study by our lab showed that HE4 overexpression in SKOV3 cells suppresses cisplatin-mediated upregulation of *EGR1* [9]. In the present study, we observed that HE4 downregulated *EGR1* expression, which is consistent with these previous results. Conversely, BCI treatment opposed the effect of rHE4 on *EGR1* expression. c-*JUN*, which is also an ERK responsive gene, was regulated in the opposite direction as *EGR1*. rHE4 treatment upregulated expression of c-*JUN*, which is consistent with its role as a promoter of tumor growth and proliferation [6, 7, 13, 14, 16]. Meanwhile, BCI again opposed this effect in BCI and rHE4 co-treated cells. Furthermore, BCI suppressed chemotherapy-mediated increases in c-*JUN* levels. The effects of BCI on *EGR1* and c-*JUN* together may contribute to the overall increased efficacy of BCI and chemotherapy treatment over chemotherapy alone, and could explain the opposing effects of BCI and HE4 on chemotherapy response.

The pathway focused qPCR array revealed further differences in gene expression profiles between the different treatment groups. As expected, *ELK1*, an ERK response gene that encodes for a transcription factor that regulates ERK response genes such as *EGR1*, was upregulated with BCI treatment, as well as carboplatin alone. Co-treatment with BCI and carboplatin resulted in similar expression profiles as either treatment alone, save for a robust decrease in *ERBB3* gene expression with co-treatment compared to carboplatin treatment alone, suggesting that downregulation of *ERBB3* may also be involved in the increased efficacy of co-treatment. *ERBB3* (HER3) is a type 1 receptor tyrosine kinase (RTK) that lacks intrinsic kinase function, and must dimerize with another ERBB receptor—particularly ERBB2 (HER2)—to activate signaling. ERBB3 is as a potent partner for and may even be required to maintain the oncogenic activity of ERBB2, which has no known ligand [34–41]. Upon ERBB3 ligand binding and subsequent hetero-dimerization with ERBB2, diverse signaling pathways may be activated, such as the PI3K/AKT pathway, which is heavily involved in promoting chemoresistance in many cancers [42–44]. Most importantly, a role for ERBB3 has been described in promoting chemoresistance and tumor progression in ovarian cancer [45–47].

In contrast to the effects of carboplatin or BCI on SKOV3 cells, paclitaxel elicited a markedly different expression profile. Combinatorial treatment with BCI and paclitaxel produced increases in BCI regulated genes such as *ELK1* and *ERBB2* compared to paclitaxel alone, and also elicited a small reduction in *ERBB3* levels as well. Therefore, it is possible that the increase in cell death elicited by co-treatment with paclitaxel and BCI may occur via the upregulation of certain BCI-regulated genes, as well as the downregulation of *ERBB3*. Further studies are required to elucidate the exact involvement of ERBB3, ELK1 and its downstream effectors such as EGR1, as well as c-JUN, on the synergistic effect of BCI on cytotoxicity. It is important to mention that BCI also possesses activity against another DUSP-family member, DUSP1 [48]. DUSP1 is a class -1 DUSP that has selectivity for JNK, ERK, and p38. DUSP1 possesses the strongest selectivity for JNK, unlike DUSP6, which only possesses substrate selectivity for ERK [49]. Therefore, we cannot discount that the activity of BCI on DUSP1 could also be influencing phospho-ERK signaling and chemoresponse. However, since we are primarily interested in the clinical relevance of a small molecule inhibitor, we chose to use BCI in our study despite this potential off target effect.

The specific role of DUSP6 in the context EOC is not well established. One report showed that DUSP6 appears to function as a tumor suppressor in EOC [25], but our results suggest the opposite effect. Therefore, further study is needed to fully elucidate the role of DUSP6 and determine if its function is context dependent. In general, DUSP6 remains an interesting protein in that it has opposing roles in different tumor types. In some cancers, it appears to act as a tumor suppressor, while in others it acts to promote tumorigenesis and aggressive behavior [20–25]. Our results are consistent with a recent study by Wu et al. (2018) showing DUSP6 involvement in cisplatin resistance in gastric cancer [23]. The authors observed an increase in phospho-ERK with BCI treatment, but a downregulation of the ERK-response genes *RPS6KA1*, *EGR1*, *MMP2*, *MMP9*, *MYC*, and *ELK3*, again showing that activation of ERK does not automatically lead to activation of its target genes. In our study, we observed different effects of DUSP6 inhibition on ERK-response genes depending upon gene function—namely, upregulation of *EGR1* and downregulation of c-*JUN*. Furthermore, Wu et al. found that BCI treatment enhanced cisplatin sensitivity in gastric cancer cells and *in vivo* xenografts. Collectively, our study and the one by Wu et al. illustrate that the relationship between ERK activation and downstream gene activation is not straightforward, and appears to be highly context-dependent. Therefore, although BCI serves to increase ERK activation and increase HE4 levels, it has differing effects on ERK response genes, which can potentially be manipulated to enhance chemotherapy efficacy.

In conclusion, this study highlights a novel function of DUSP6 in EOC, and reveals that it may be involved in regulating chemoresponse. Targeting HE4 and/or DUSP6 in EOC may be an effective method of reversing chemoresistance and improving long-term response rates in selected patient populations.

## MATERIALS AND METHODS

### Cell culture and treatments

SKOV3 and OVCAR8 cells were obtained from American Type Culture Collection (ATCC) and kept at low passage in Dulbecco Modified Eagle Medium (DMEM) with 10% fetal bovine serum and 1% penicillin/streptomycin, in a humidified incubator at 37°C/5% CO_2_. Cells were plated at sub-confluent density the day before treatments. Cells were treated with 3.75 µM BCI (Sigma Aldrich, B4313), 20 nM recombinant HE4 (My BioSource, MBS355616 or Raybiotech, 230-30001-10), 100 µM carboplatin (Sigma Aldrich, C2538), 10 nM paclitaxel (Sigma Aldrich, T7402), or control treatments (0.037% DMSO and/or H_2_0) for indicated time points. SKOV3-NV, SKOV3-C1, and SKOV3-C1-CRISPR cell lines were generated as previously described [9, 14].

### Western Blot

Western blot was performed as previously described [10]. GAPDH was used as a loading control. Antibodies and dilutions used are as follows:

MKP-3 (DUSP6) (Santa Cruz, sc-377070; 1:100 or Novus Biologicals, NBP2-67320, 1:50)

HE4 (Origene, TA307787, 1:2000)

GAPDH (Cell Signaling, 2118, 1:2000)

Phospho-p44/42 (ERK1/2) (Cell Signaling, 9101, 1:2000)

### Densitometry

Image J “analyze gel” function was used to perform densitometry analysis of western blot images in 8-bit TIFF format. Band densities were normalized to GAPDH, and the control was set to 1 for plotted graphs.

### Quantitative RT-PCR

Quantitative RT-PCR was performed as previously described [10]. Validated primers for *DUSP6*, *EGR1*, c-*JUN*, and *ERBB3* were purchased from https://www.realtimeprimers.com. Custom primer sequences (Invitrogen) are as follows:

18s rRNA (F) – CCG CGG TTC TAT TTT GTT GG

18s rRNA (R) – GGC GCT CCC TCT TAA TCA TG

HE4 (F) – CTG CCC CCA GGT GAA CAT TA

HE4 (R) – CCA TTG CGG CAG CAT TTC AT

### qPCR Array

One set of SKOV3 cell RNA from the experiment in Figure 3 was used for the qPCR array (control, BCI, carboplatin, BCI+carboplatin, paclitaxel, and BCI+paclitaxel). RNA was reverse transcribed using the RT2 First Strand Kit (Qiagen, 330401), and cDNA was then added with SYBR green mastermix (Qiagen, 330504) to 96-well RT2 Profiler Human Cancer Drug Resistance PCR Array plates (Qiagen, PAHS-004Z), according to the manufacturer’s instructions. The plates were run on an ABI 7500 qPCR machine, with 10 min at 95 °C, and 40 cycles of 15s at 95 °C and 1 min at 60 °C. An automated baseline was used, with thresholds manually adjusted to remain constant across all plates. Data was analyzed using the GeneGlobe Data Analysis Center at https://www.qiagen.com. The housekeeping gene RPLP0 was used for normalization, which was automatically selected by the software based on stable expression between treatments. Calculations of relative expression levels were performed using the using the ΔΔCt method All samples passed quality control standards (array reproducibility, RT efficiency, and genomic DNA contamination).

### Cell viability assay

Cells were seeded at 2000 cells/well in 96-well plates, and treated as described above. After 24 h, cell viability assays were performed by adding 10 µl/well of CellTiter 96^®^ Aqueous One Solution Cell Proliferation MTS Assay (Promega, G3580), incubating at 37°C/5% CO^2^ for 2 h, and reading absorbance at 492 nm. Results are displayed as percent survival relative to vehicle treated cells.

### Immunohistochemistry

Immunohistochemical staining of an ovarian cancer microarray (US Biomax, OV802a) was performed as previously described [50], using antibodies for HE4 (Santa Cruz, sc-293473) and DUSP6 (MyBioSource, MBS8516662). Confocal microscopy was performed by an independent imaging technician at the Rhode Island Hospital Digital Imaging Core Facility with a Nikon C1si confocal (Nikon Inc. Mellville, NY, USA). Two to three fields/sample were randomly selected based on DAPI staining, and minimum, mean, and maximum gray values (pixels) were determined for each field. For the tumor microarray, normal adjacent tissues were used to set the threshold for positive staining. Integrated optical density (IOD) was calculated in serous samples using the mean values multiplied by the total area.

### Statistics

Where statistics are shown, n≥3 biological replicates. p-values from quantitative PCR, MTS assay, and western blots were determined by unpaired, 1-tailed Student *t*-test. For correlation analysis, Spearman rank test was used to determine R value and corresponding p-values using the calculators at https://www.socscistatistics.com. Differences were considered statistically significant when p < 0.05.

## SUPPLEMENTARY MATERIALS FIGURE AND DATASET





## Abbreviations

DUSP6: dual specificity phosphatase 6
ERK: extracellular signal regulated kinase
HE4: human epididymis protein 4
rHE4: recombinant HE4
EOC: epithelial ovarian cancer
CA 125: cancer antigen 125
BCI: (E)-2-Benzylidene-3-(cyclohexylamino)-2,3-dihydro-1H-inden-1-one
EGR1: early growth response 1
IOD: integrated optical density
NAT: normal adjacent tissue
DMEM: dulbecco’s modified eagle medium
DMSO: dimethyl sulfoxide
MTS: [3-(4,5-dimethylthiazol-2-yl)-5-(3-carboxymethoxyphenyl)-2-(4-sulfophenyl)-2H-tetrazolium
