# Supplementary material for: Inhibition of DUSP6 sensitizes ovarian cancer cells to chemotherapeutic agents via regulation of ERK signaling response genes
Source: Oncotarget. 2019 May 21;10(36):3315–27. (PMC6534361)
Supplement: Supplementary file 1 [file oncotarget-10-3315-s001.pdf]

Inhibition of DUSP6 sensitizes ovarian cancer cells to chemotherapeutic agents via regulation of ERK signaling response genes

SUPPLEMENTARY MATERIALS

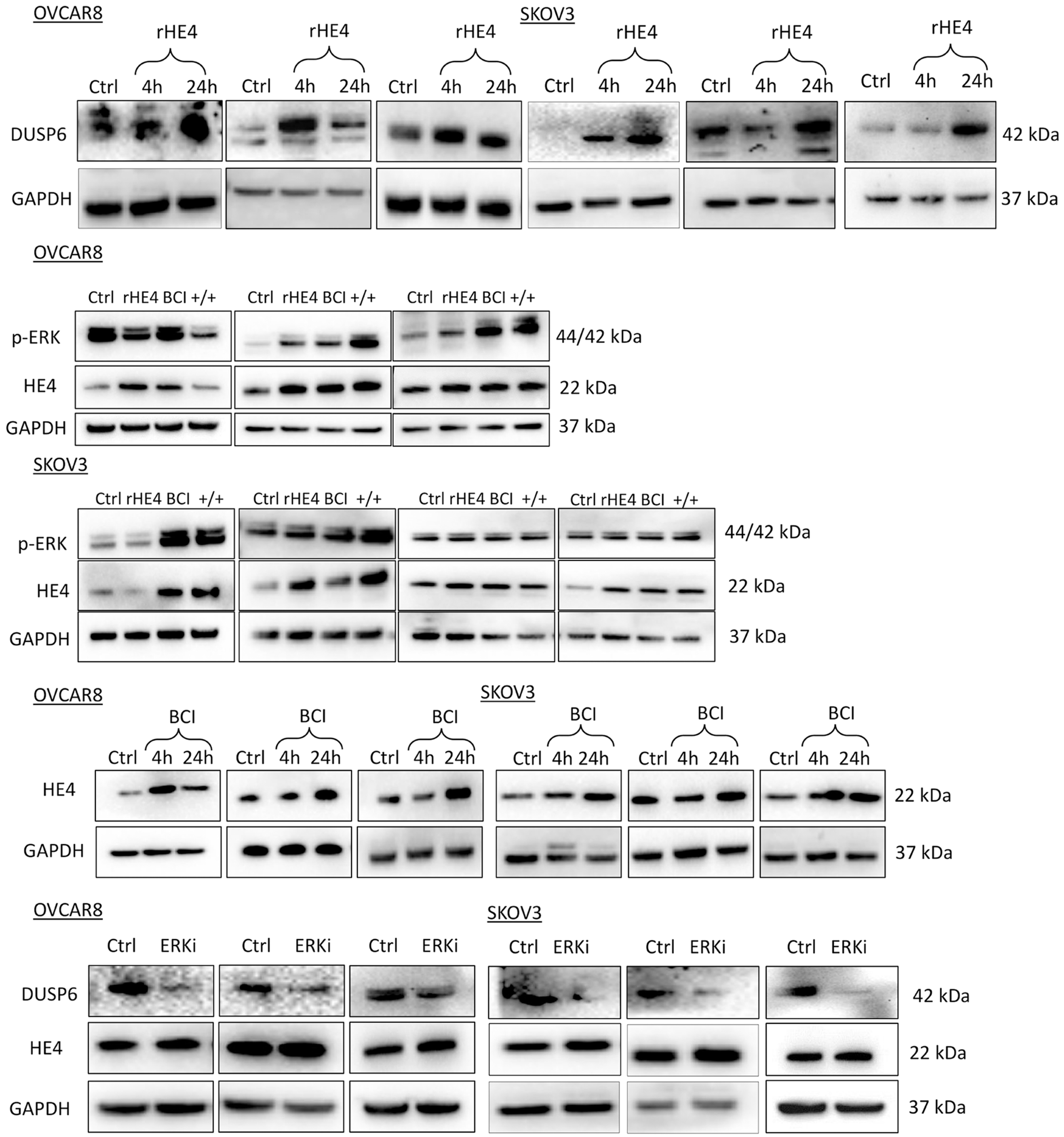

Supplementary Figure 1: Triplicate western blots from Figure 1.

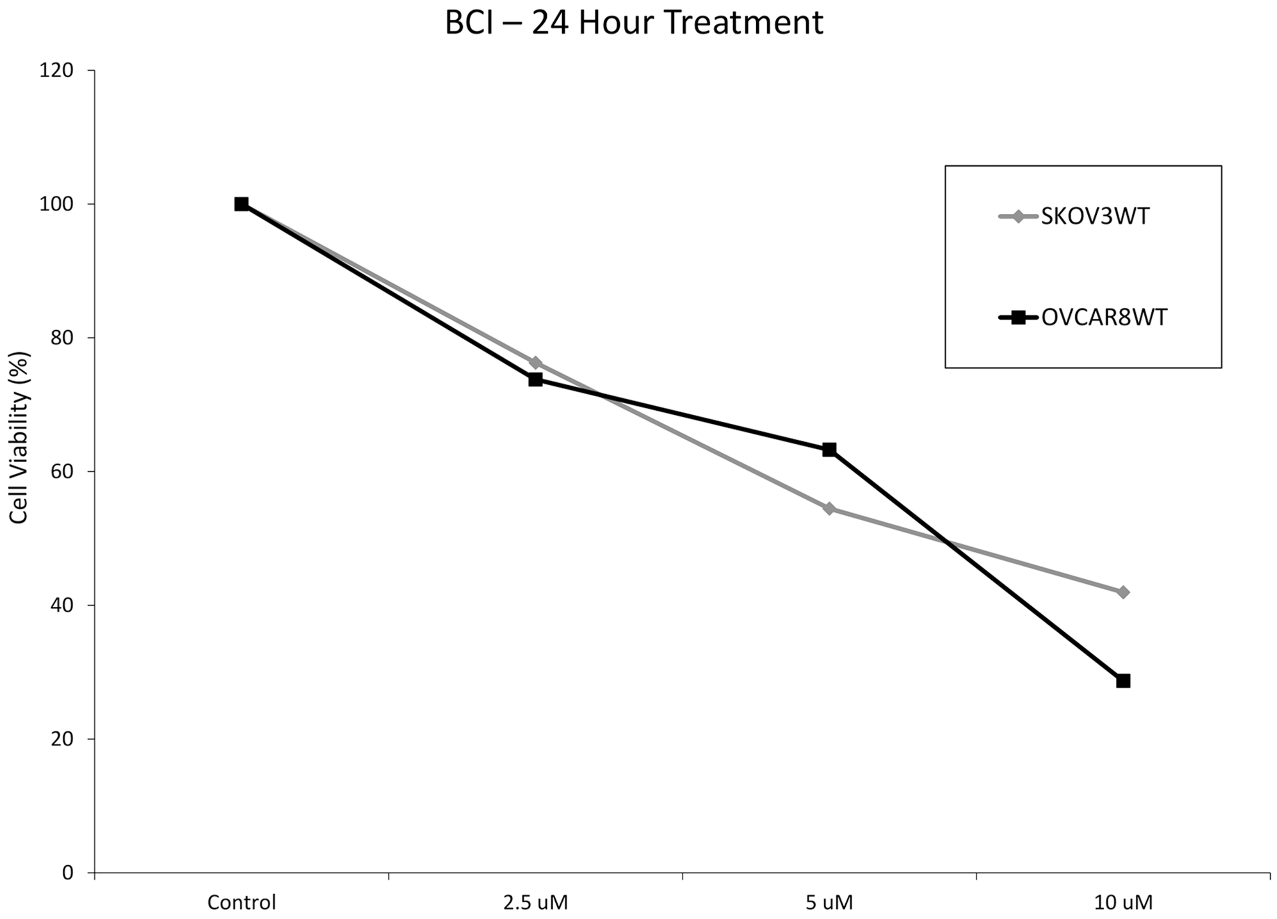

Supplementary Figure 2: BCI dose curve. OVCAR8 (black line) and SKOV3 (gray line) cells were treated with various doses of BCI for 24 h and cell viability was measured with MTS assay.

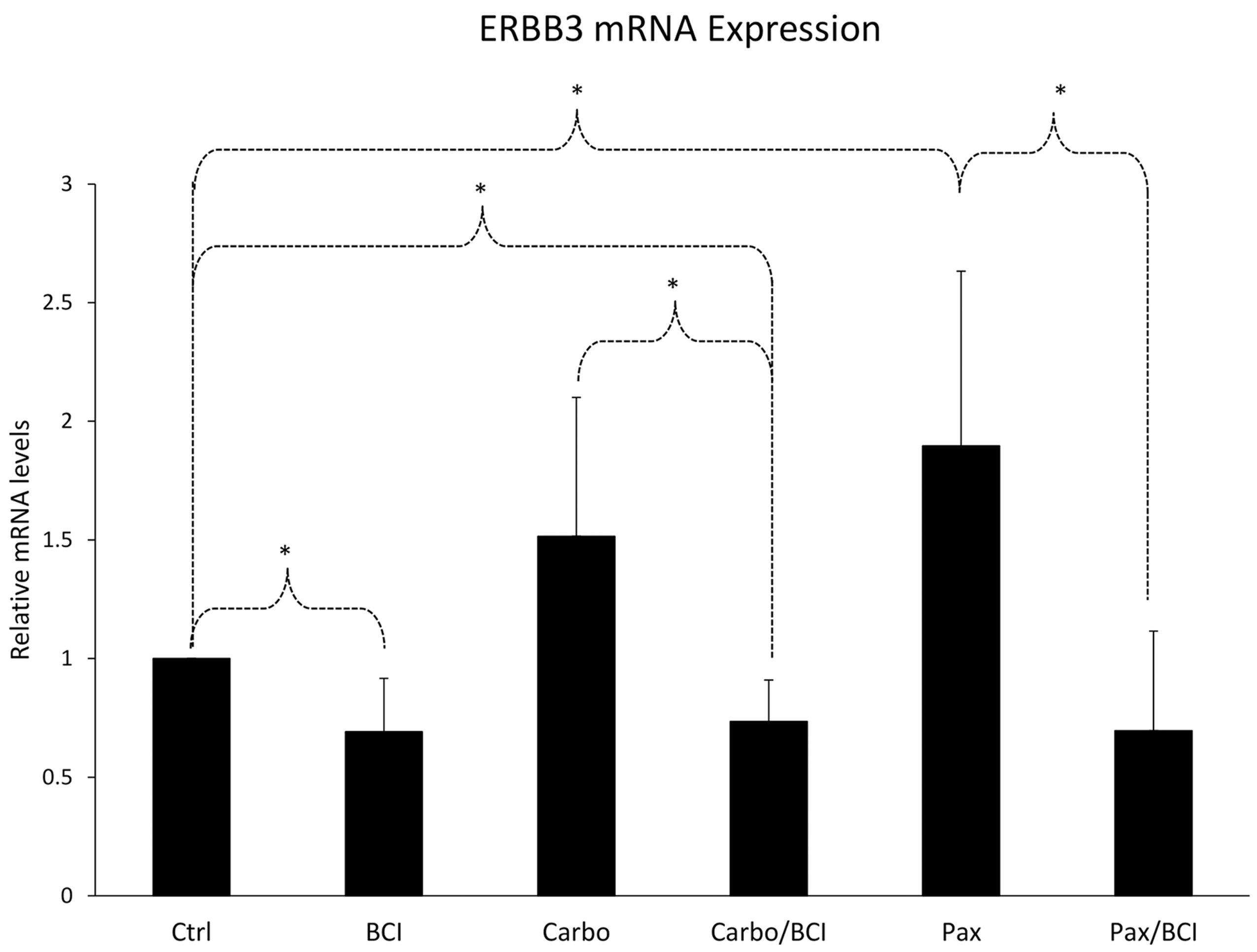

Supplementary Figure 3: BCI suppresses ERBB3 mRNA expression in OVCAR8 cells. OVCAR8 cells were treated with 3.75 µM BCI, 10 nM paclitaxel, 100 µM carboplatin, or BCI in combination with paclitaxel or carboplatin for 24 h, and *ERBB3* mRNA levels were measured by qPCR. Error bars represent standard deviation of three biological replicates. *$p<0.05$.

Supplementary Dataset 1: Quantitative PCR pathway focused array raw data.

See Supplementary File 1
